# Pediatric Gastrointestinal Endoscopy at a Tertiary Care Hospital: A Two-Year Analysis of Procedures and Endoscopic Findings

**DOI:** 10.7759/cureus.113455

**Published:** 2026-07-27

**Authors:** Carlos Alexis Mascote Rodriguez, Yoeli Escandon-Espinoza, Katia Lopez Garcia

**Affiliations:** 1 Gastrointestinal Endoscopy, Hospital Regional de Alta Especialidad Bicentenario de la Independencia, Instituto de Seguridad y Servicios Sociales de los Trabajadores del Estado (ISSSTE), Tultitlan, MEX

**Keywords:** colonoscopy, endoscopic finding, esophagogastroduodenoscopy, pediatric endoscopy, pediatric gastroenterology, tertiary care referral hospital

## Abstract

Background: Gastrointestinal endoscopy has become an essential diagnostic and therapeutic modality in pediatric patients. Despite its widespread use, reports describing the experience of tertiary care centers, particularly in Latin America, remain scarce. This study aimed to characterize the demographic profile, clinical indications, endoscopic procedures, endoscopic findings, and histopathological outcomes of pediatric patients undergoing gastrointestinal endoscopy at a tertiary care hospital.

Methods: A retrospective, observational, descriptive study was conducted including all gastrointestinal endoscopic procedures performed in patients younger than 18 years of age over a two-year period. Demographic characteristics, type of procedure, clinical indications, endoscopic findings, therapeutic interventions, diagnostic yield, and histopathological results were collected from electronic medical records and endoscopy reports. Descriptive statistical analysis was performed.

Results: A total of 88 endoscopic procedures were performed in 77 pediatric patients, with a mean age of 12.18 years (range: eight months to 17 years). Male patients accounted for 64 of 88 procedures (72.7%). Esophagogastroduodenoscopy (EGD) was the most frequently performed procedure (71/88, 80.7%), followed by colonoscopy (15/88, 17%) and endoscopic retrograde cholangiopancreatography (ERCP) (2/88, 2.3%). The leading indications were gastroesophageal reflux disease (GERD), lower gastrointestinal bleeding, and foreign body ingestion. Clinically significant endoscopic findings were identified in 76 procedures (86.3%), with gastropathy, esophagitis, and foreign bodies representing the most common diagnoses. Tissue biopsies were obtained in 43 procedures (48.9%), and histopathological abnormalities were identified in 36 of 43 (83.7%) biopsy specimens, predominantly chronic gastritis and *Helicobacter pylori* infection.

Conclusions: Gastrointestinal endoscopy demonstrated a high diagnostic yield in pediatric patients managed at a tertiary care hospital. Upper gastrointestinal inflammatory disorders represented the predominant endoscopic findings, while foreign body ingestion remained one of the leading indications in younger children. These findings provide an overview of the spectrum of pediatric gastrointestinal diseases encountered in a tertiary referral center and support the role of gastrointestinal endoscopy as a safe and effective diagnostic tool in this population.

## Introduction

Gastrointestinal endoscopy has undergone remarkable advances over the past several decades, becoming an indispensable diagnostic and therapeutic modality in pediatric gastroenterology. The development of smaller-caliber endoscopes, dedicated pediatric accessories, and advances in sedation and anesthesia have enabled the safe performance of endoscopic procedures across all pediatric age groups, substantially expanding both diagnostic and therapeutic capabilities [1].

Currently, gastrointestinal endoscopy plays a central role in the evaluation and management of a broad spectrum of pediatric gastrointestinal disorders, including inflammatory bowel disease, eosinophilic gastrointestinal diseases, gastrointestinal bleeding, foreign body ingestion, congenital anomalies, pancreaticobiliary disorders, and other organic diseases affecting the digestive tract. Beyond diagnosis, pediatric endoscopy also enables minimally invasive therapeutic interventions with excellent efficacy and safety when performed by experienced endoscopists following standardized quality indicators [2,3].

The indications for esophagogastroduodenoscopy (EGD) and colonoscopy in children are generally comparable to those in adults; however, pediatric patients present unique clinical challenges. Younger children frequently cannot accurately describe their symptoms, making careful clinical assessment essential when selecting candidates for endoscopic evaluation. Current international guidelines recommend endoscopy for patients presenting with alarm symptoms, gastrointestinal bleeding, dysphagia, persistent vomiting, unexplained anemia, foreign body ingestion, caustic ingestion, chronic diarrhea, inflammatory bowel disease, colorectal polyps, and other conditions requiring diagnostic or therapeutic intervention [3,4].

Previous studies evaluating pediatric endoscopy have consistently demonstrated that inflammatory upper gastrointestinal disorders, particularly gastritis, gastropathy, esophagitis, and duodenitis, represent the most common endoscopic findings. Furthermore, the diagnostic yield of gastrointestinal endoscopy is highly dependent on appropriate patient selection and adherence to evidence-based clinical indications [5-8].

In recent years, advanced endoscopic procedures have further expanded the scope of pediatric gastroenterology. Endoscopic retrograde cholangiopancreatography (ERCP), endoscopic ultrasound (EUS), and capsule endoscopy have demonstrated favorable safety and efficacy profiles in carefully selected pediatric patients, particularly for pancreaticobiliary disorders and small bowel diseases. Current international guidelines recommend reserving ERCP primarily for therapeutic indications after adequate noninvasive imaging has established the diagnosis [4,9,10].

Despite the increasing use of pediatric gastrointestinal endoscopy worldwide, reports describing institutional experience in tertiary care hospitals remain limited, particularly in developing countries. Characterizing local disease patterns, procedural indications, endoscopic findings, and histopathological outcomes is essential for optimizing patient selection, improving resource allocation, and establishing quality indicators for pediatric endoscopy services. Therefore, the aim of the present study was to describe the demographic characteristics, clinical indications, procedural profile, endoscopic findings, diagnostic yield, and histopathological outcomes of pediatric patients undergoing gastrointestinal endoscopy at a tertiary care hospital over a two-year period.

## Materials and methods

Study design

A retrospective, observational, descriptive, single-center study was conducted at the Gastrointestinal Endoscopy Unit of Hospital Regional de Alta Especialidad Bicentenario de la Independencia, Instituto de Seguridad y Servicios Sociales de los Trabajadores del Estado (ISSSTE), a tertiary care hospital in Tultitlan, Mexico. The study included all pediatric patients who underwent gastrointestinal endoscopic procedures between January 2024 and January 2026.

At our institution, pediatric patients were referred for gastrointestinal endoscopy by pediatric gastroenterologists or treating physicians according to the clinical indications and institutional practice, following current guideline-based recommendations.

Study population

The study population consisted of consecutive pediatric patients younger than 18 years who underwent gastrointestinal endoscopy at our institution between January 2024 and January 2026. Eligible procedures included EGD, colonoscopy, and ERCP. Consecutive eligible procedures were retrospectively identified from the institutional endoscopy database. Only procedures with complete endoscopic reports and corresponding clinical records available for review were included in the final analysis.

Patients with incomplete endoscopic reports, inadequate bowel preparation preventing complete colonoscopic evaluation, insufficient clinical information, canceled or incomplete procedures, and neonatal patients were excluded from the study. Neonates requiring gastrointestinal endoscopy were routinely referred to another specialized institution because appropriate neonatal endoscopic equipment was unavailable at our center.

Data collection

Clinical and procedural data were obtained through retrospective review of electronic medical records, endoscopy reports, and histopathological reports. Demographic variables included age, sex, and age group. Procedural variables included the type of endoscopic procedure, clinical indication, primary endoscopic finding, therapeutic intervention, and histopathological diagnosis when biopsy specimens were obtained.

Patients were classified into four age groups according to pediatric age categories: infants (<2 years), preschool children (2-5 years), school-aged children (6-11 years), and adolescents (12-17 years). Endoscopic examinations were categorized as either normal or abnormal according to the presence of clinically relevant endoscopic findings documented in the procedure report. For descriptive analysis, the endoscopic category "gastropathy" included nonspecific gastric mucosal abnormalities reported in the original endoscopy reports, such as erosive, erythematous, and edematous gastropathy. Because individual subtypes were infrequent and this was a retrospective descriptive study, they were analyzed as a single category to facilitate data presentation.

For patients undergoing gastric biopsy because of endoscopic gastropathy, tissue samples were obtained according to the Updated Sydney protocol. Histopathological diagnoses, including chronic gastritis and *Helicobacter pylori *infection, were recorded from the final pathology reports as originally reported, without further reclassification because of the retrospective nature of the study.

Because several patients underwent more than one endoscopic procedure during the study period, each procedure was analyzed as an independent observational unit.

Outcome measures

The primary objective of the study was to describe the demographic characteristics, clinical indications, procedural profile, endoscopic findings, diagnostic yield, and histopathological outcomes of pediatric gastrointestinal endoscopy performed at our institution over a two-year period.

Secondary objectives included describing the distribution of endoscopic procedures according to age group, determining the frequency of abnormal endoscopic findings and histopathological diagnoses, and evaluating the relationship between patient age and the spectrum of gastrointestinal diseases identified during endoscopic evaluation.

Statistical analysis

Continuous variables are presented as mean±standard deviation (SD) or median with range, as appropriate. Categorical variables are expressed as absolute frequencies and percentages. Because of the descriptive nature of the study, no inferential statistical analyses were performed. Data were analyzed using Microsoft Excel® Version 16.77 (Microsoft Corporation, Redmond, Washington, United States).

Ethical considerations

The study was conducted in accordance with the ethical principles of the Declaration of Helsinki and institutional regulations. Given the retrospective nature of the study and the use of anonymized clinical data, the requirement for informed consent was waived by the institutional review board. Patient confidentiality was maintained throughout the study, and no identifiable personal information was collected or disclosed.

## Results

Patient characteristics

A total of 88 gastrointestinal endoscopic procedures were performed in 77 pediatric patients during the study period. The mean patient age was 12.18 years (range: eight months to 17 years), with adolescents representing the largest age group. Male patients accounted for 64 of 88 procedures (72.7%). Detailed demographic characteristics are presented in Table 1.

**Table 1:** Baseline characteristics of the study population

Characteristic	Result
Patients, n	77
Procedures, n	88
Age, mean±SD (years)	12.18±5.56
Median age (years)	14
Age range	8 months to 17 years
Sex	
Male	64 (72.7%)
Female	24 (27.3%)
Age group	
Infants	6 (6.8%)
Preschool children	12 (13.6%)
School-aged children	10 (11.3%)
Adolescents	60 (68.1%)
Biopsies obtained	43 (48.9%)

Endoscopic procedures

EGD was the most commonly performed procedure, accounting for 71 of 88 procedures (80.7%) of all endoscopic examinations. Colonoscopy represented 15 of 88 procedures (17%), while ERCP accounted for two of 88 procedures (2.3%). The distribution of endoscopic procedures is summarized in Table 2.

**Table 2:** Distribution of gastrointestinal endoscopic procedures Each endoscopic procedure was analyzed as an independent event.

Procedure	n (%)
Esophagogastroduodenoscopy	71 (80.7%)
Colonoscopy	15 (17%)
Endoscopic retrograde cholangiopancreatography	2 (2.3%)
Total	88 (100%)

Indications for endoscopy

The most frequent indication for gastrointestinal endoscopy was gastroesophageal reflux disease (GERD), followed by lower gastrointestinal bleeding and foreign body ingestion. Less common indications included dysphagia, chronic diarrhea, constipation, persistent vomiting, caustic ingestion, and evaluation of inflammatory bowel disease. The complete distribution of clinical indications is shown in Table 3.

**Table 3:** Clinical indications for gastrointestinal endoscopy

Clinical indication	n (%)
Gastroesophageal reflux disease	15 (17%)
Foreign body ingestion	14 (15.9%)
Lower gastrointestinal bleeding	14 (15.9%)
Dysphagia	12 (13.6%)
Upper gastrointestinal bleeding	11 (12.5%)
Dyspepsia	7 (8%)
Caustic ingestion	4 (4.5%)
Hiccups	3 (3.4%)
Choledocholithiasis	2 (2.3%)
Persistent vomiting	1 (1.1%)
Constipation	1 (1.1%)
Early satiety	1 (1.1%)
Irritable bowel syndrome	1 (1.1%)
Pre-bariatric evaluation	1 (1.1%)
Hemolacria	1 (1.1%)
Total	88 (100%)

Endoscopic findings

Clinically significant endoscopic findings were identified in 76 of the 88 procedures (86.3%), while 12 examinations (13.7%) were reported as normal. Gastropathy was the most frequent finding, followed by esophagitis and foreign body detection. Additional findings included duodenitis, colonic polyps, inflammatory bowel disease, erosive lesions, vascular abnormalities, and other less frequent gastrointestinal disorders. The distribution of endoscopic findings is summarized in Table 4.

**Table 4:** Endoscopic findings Percentages were calculated using the total number of endoscopic procedures (n=88). Procedures with normal endoscopic examinations were excluded from this table. *Abnormal gastroesophageal flap valve was defined as a Hill grade III or IV gastroesophageal flap valve observed during upper endoscopy.

Endoscopic finding	n (%)
Gastropathy	19 (21.6%)
Esophagitis	14 (15.9%)
Foreign body	10 (11.4%)
Esophageal stricture	5 (5.7%)
Colonic polyp	4 (4.5%)
Duodenitis	2 (2.3%)
Hiatal hernia	2 (2.3%)
Proctitis	2 (2.3%)
Bile duct dilatation	2 (2.3%)
Abnormal gastroesophageal flap valve (Hill grade III/IV)*	1 (1.1%)
Mucosal tear	1 (1.1%)
Colitis	1 (1.1%)
Gastric ulcer	1 (1.1%)
Duodenal dilatation	1 (1.1%)
Hemorrhoidal disease	1 (1.1%)
Pharyngeal erythema	1 (1.1%)
Intestinal metaplasia	1 (1.1%)
Colorectal tumor	1 (1.1%)

Endoscopic findings according to age group

The spectrum of endoscopic findings varied according to patient age. Inflammatory disorders of the upper gastrointestinal tract, including gastropathy and esophagitis, predominated among adolescents, whereas foreign body ingestion was observed mainly in younger children. Colonoscopic abnormalities were more frequently identified in school-aged children and adolescents. The age-specific distribution of endoscopic findings is detailed in Table 5.

**Table 5:** Distribution of primary endoscopic findings according to age group *Inflammatory upper gastrointestinal disorders: gastropathy, esophagitis, duodenitis, and gastric ulcer. **Structural abnormalities: esophageal stricture, hiatal hernia, and enlarged hiatal opening. ***Colorectal disorders: colonic polyps, proctitis, colitis, and colorectal tumor. ****Pancreatobiliary disorders: bile duct dilatation. *****Other findings: uncommon endoscopic abnormalities not included in the previous categories.

Primary endoscopic finding	Infants	Preschool children	School-aged children	Adolescents
Inflammatory upper gastrointestinal disorders*	2	2	4	28
Foreign body	2	4	3	1
Structural abnormalities**	0	1	0	7
Colorectal disorders***	1	1	0	6
Pancreatobiliary disorders****	0	0	0	2
Other findings*****	0	1	0	5

Histopathological findings

Biopsies were obtained in 43 procedures (48.9%). Histopathological abnormalities were identified in 36 of 43 biopsy specimens (83.7%). Chronic gastritis was the most common diagnosis (13/43, 30.2%), followed by *Helicobacter pylori *infection (11/43, 25.6%). A detailed summary of histopathological diagnoses is presented in Table 6.

**Table 6:** Histopathological findings in biopsied specimens *Percentages were calculated using the total number of biopsied procedures (n=43). Multiple histopathological diagnoses could be identified in a single biopsy specimen; therefore, the sum of individual findings exceeds the total number of biopsied procedures. Only patients who underwent tissue biopsy were included in this analysis.

Histopathological finding	n (%)
Chronic gastritis	13 (30.2%)
*Helicobacter pylori* infection	11 (25.6%)
Chronic colitis	3 (7%)
Esophagitis	3 (7%)
Colorectal polyps	3 (7%)
Normal histopathology	5 (11.6%)
Lymphoid hyperplasia	1 (2.3%)
Dysplasia	1 (2.3%)
Pseudomembranous colitis	1 (2.3%)

Overall, gastrointestinal endoscopy demonstrated a high diagnostic yield, with clinically significant findings identified in more than four-fifths of all procedures performed during the study period. Upper gastrointestinal inflammatory disorders accounted for the majority of abnormal examinations, whereas therapeutic interventions were primarily performed in patients undergoing foreign body extraction and ERCP.

## Discussion

Gastrointestinal endoscopy has become an essential component of pediatric gastroenterology, providing accurate diagnosis and minimally invasive treatment for a broad spectrum of gastrointestinal diseases. Continuous advances in endoscopic technology, pediatric anesthesia, and quality standards have substantially improved the safety and effectiveness of these procedures, allowing their routine implementation even in very young children. The present study describes the experience of a tertiary referral center over a two-year period and provides a comprehensive characterization of pediatric gastrointestinal endoscopy in routine clinical practice [1-3,9].

EGD represented more than four-fifths of all procedures performed, whereas colonoscopy and ERCP accounted for a considerably smaller proportion. This distribution is consistent with previous pediatric series, in which upper gastrointestinal symptoms constitute the principal indication for referral to endoscopy units. Likewise, current international guidelines recognize EGD as the most frequently performed pediatric endoscopic procedure because of its broad diagnostic applicability and therapeutic potential [3-5].

The most common indications identified in our study were GERD, foreign body ingestion, and lower gastrointestinal bleeding. These findings are comparable to those reported in previous observational studies, although the relative frequency of individual indications varies according to institutional referral patterns, healthcare systems, and the characteristics of the population evaluated. As a tertiary referral hospital, our institution receives patients with a high pretest probability of organic gastrointestinal disease, which likely contributed to the elevated diagnostic yield observed in our cohort [5-8].

Clinically significant endoscopic abnormalities were identified in 76 of 88 procedures (86.3%), demonstrating that appropriate patient selection resulted in a high diagnostic yield. Gastropathy and esophagitis represented the predominant endoscopic findings, supporting previous reports in which inflammatory disorders of the upper gastrointestinal tract constitute the most common diagnoses in pediatric endoscopy. Foreign body ingestion remained one of the leading findings among younger children, reflecting the well-established epidemiology of accidental ingestion during early childhood [5-8].

Age-specific analysis demonstrated distinct patterns of gastrointestinal disease. Inflammatory upper gastrointestinal disorders predominated among adolescents, whereas foreign body ingestion occurred mainly in infants and preschool children. Structural abnormalities and colorectal disorders were identified predominantly in older children and adolescents. These observations emphasize the importance of considering patient age during the diagnostic evaluation and support age-oriented selection of endoscopic procedures [6-8].

Although ERCP accounted for only two procedures, its inclusion illustrates the availability of advanced therapeutic endoscopy within our institution. As recommended by current international guidelines, ERCP was reserved exclusively for carefully selected therapeutic indications after appropriate clinical and imaging evaluation, reflecting contemporary pediatric endoscopic practice [4,10].

Histopathological evaluation provided clinically relevant complementary information in 43 of 88 procedures (48.9%), highlighting the additional diagnostic value of tissue sampling when clinically indicated. Chronic gastritis represented the most frequent histopathological finding, followed by *Helicobacter pylori *infection. Because multiple histopathological diagnoses were recorded when present, tissue analysis allowed a more comprehensive characterization of mucosal disease than would have been possible using a single principal diagnosis. These findings reinforce current recommendations supporting targeted biopsy acquisition whenever clinically indicated, even in the presence of subtle endoscopic abnormalities [11].

One of the strengths of this study is that each endoscopic procedure was analyzed as an independent event, allowing a comprehensive description of procedural indications, diagnostic yield, endoscopic findings, and histopathological outcomes. Furthermore, the inclusion of both diagnostic and therapeutic procedures provides a realistic overview of pediatric gastrointestinal endoscopy performed in a tertiary referral center.

Nevertheless, several limitations should be acknowledged. The retrospective design depends on the accuracy and completeness of medical records, and the single-center setting may limit the generalizability of the findings. In addition, our institution does not currently possess endoscopic equipment specifically designed for neonatal patients; therefore, neonates requiring gastrointestinal endoscopy were referred to another specialized center, resulting in the underrepresentation of this age group. Despite these limitations, this study provides valuable epidemiological information and establishes a local benchmark for future pediatric endoscopy research and quality improvement initiatives. Additionally, because the total pediatric outpatient and inpatient population served during the study period was not available, the institutional burden and procedural rates could not be estimated.

## Conclusions

Pediatric gastrointestinal endoscopy showed a high diagnostic yield and served as an effective diagnostic modality across a broad range of clinical indications in our institution. EGD was the most frequently performed procedure, whereas GERD, foreign body ingestion, and lower gastrointestinal bleeding represented the leading indications for endoscopic evaluation.

Inflammatory disorders of the upper gastrointestinal tract constituted the predominant endoscopic and histopathological findings, while clinically relevant differences in disease distribution were observed according to patient age. These results highlight the importance of age-specific clinical assessment and appropriate patient selection when considering gastrointestinal endoscopy in pediatric patients.

This study provides one of the few contemporary descriptions of pediatric gastrointestinal endoscopy from a tertiary referral center in Mexico and contributes valuable epidemiological data regarding procedural characteristics, diagnostic yield, and disease patterns. These findings may serve as a reference for future multicenter studies and quality improvement initiatives in pediatric gastrointestinal endoscopy.
